# Effects of an Amino Acid-Based Formula Supplemented with Two Human Milk Oligosaccharides on Growth, Tolerability, Safety, and Gut Microbiome in Infants with Cow’s Milk Protein Allergy

**DOI:** 10.3390/nu14112297

**Published:** 2022-05-30

**Authors:** Michael S. Gold, Patrick J. Quinn, Dianne E. Campbell, Jane Peake, Joanne Smart, Marnie Robinson, Michael O’Sullivan, Josef Korbinian Vogt, Helle Krogh Pedersen, Xiaoqiu Liu, Elham Pazirandeh-Micol, Ralf G. Heine

**Affiliations:** 1 Department of Allergy & Immunology, Women’s and Children’s Hospital, University of Adelaide, Adelaide, SA 5006, Australia; patrick.quinn@adelaide.edu.au; 2 Department of Allergy & Clinical Immunology, Children’s Hospital at Westmead, University of Sydney, Sydney, NSW 2145, Australia; dianne.campbell1@health.nsw.gov.au; 3 Queensland Paediatric Immunology and Allergy Service, Queensland Children’s Hospital, University of Queensland, South Brisbane, QLD 4101, Australia; Jane.Peake@health.qld.gov.au; 4 Paediatric Allergy Services, Epworth Hospital, Richmond, VIC 3121, Australia; joanne.smart@rch.org.au; 5 Melbourne Allergy Centre & Children’s Specialists Medical Group, Parkville, VIC 3152, Australia; marnie.robinson@hotmail.com; 6 Department of Immunology, Perth Children’s Hospital, Nedlands, WA 6009, Australia; 7 Clinical Microbiomics, DK-2100 Copenhagen, Denmark; josef@clinical-microbiomics.com (J.K.V.); helle@clinical-microbiomics.com (H.K.P.); 8 Biostatistics and Data Science Division, The George Institute for Global Health, University of New South Wales, Sydney, NSW 2042, Australia; xliu@georgeinstitute.org.au; 9 Nestlé Health Science, CH-1800 Vevey, Switzerland; elham.pazirandehmicol@nestle.com (E.P.-M.); ralf.heine@au.nestle.com (R.G.H.)

**Keywords:** 2′-fucosyllactose, lacto-N-neotetraose, hypoallergenic formula, gut microbiome, metagenomic sequencing, bifidobacteria, dysbiosis, short chain fatty acids

## Abstract

This open-label, non-randomized, multicenter trial (Registration: NCT 03661736) aimed to assess if an amino acid-based formula (AAF) supplemented with two human milk oligosaccharides (HMO) supports normal growth and is well tolerated in infants with a cow’s milk protein allergy (CMPA). Term infants aged 1–8 months with moderate-to-severe CMPA were enrolled. The study formula was an AAF supplemented with 2′-fucosyllactose (2′-FL) and lacto-N-neotetraose (LNnT). Infants were fed the study formula for 4 months and were offered to remain on the formula until 12 months of age. Tolerance and safety were assessed throughout the trial. Out of 32 infants (mean age 18.6 weeks; 20 (62.5%) male), 29 completed the trial. During the 4-month principal study period, the mean weight-for-age Z score (WAZ) increased from –0.31 at the baseline to +0.28 at the 4-months’ follow-up. Linear and head growth also progressed along the WHO child growth reference, with a similar small upward trend. The formula was well tolerated and had an excellent safety profile. When comparing the microbiome at the baseline to the subsequent visits, there was a significant on-treatment enrichment in HMO-utilizing bifidobacteria, which was associated with a significant increase in fecal short-chain fatty acids. In addition, we observed a significant reduction in the abundance of fecal Proteobacteria, suggesting that the HMO-supplemented study formula partially corrected the gut microbial dysbiosis in infants with CMPA.

## 1. Introduction

An amino acid-based formula (AAF) is a specialized type of hypoallergenic formula that is strictly devoid of allergenic food proteins [1]. Instead, free amino acids are used as the dietary nitrogen source [2]. In clinical practice, AAF is reserved for the nutritional management of formula-fed infants with moderate-to-severe cow’s milk protein allergy (CMPA), including those who failed to respond to a trial of an extensively hydrolyzed formula (EHF) [3,4]. In addition, there are several indications where an AAF is suitable as first-line treatment, such as anaphylaxis to cow’s milk protein (CMP), multiple food allergy of infancy, eosinophilic esophagitis, or gastrointestinal CMPA with significant growth failure [1,5].

CMPA in young infants is associated with intestinal microbial dysbiosis, characterized by the suppression of infantile bifidobacteria and the enrichment of Proteobacteria and other gram-negative gut bacteria [6]. The establishment of a healthy gut microbiome in early infancy requires the presence of human milk oligosaccharides (HMO), which provide the preferred substrate for the infantile *Bifidobacterium* species and *Bacteroides* [7,8,9,10]. Several studies have demonstrated that bifidobacteria promote early immune maturation, including the enhancement of innate and adaptive immunity to protect against infections [11,12,13].

HMO manufactured by bacterial biofermentation from lactose have recently become available as novel ingredients in infant formulas for healthy infants and those with CMPA [14,15,16]. The concentrations of HMO in breast milk vary significantly between mothers, as well as for stages of lactation and maternal Lewis secretor status [17]. The concentrations of the HMO, 2′-fucosyllacose (2′-FL) at 1 g/L and lacto-N-neotetraose (LNnT) at 0.5 g/L in the study formula were chosen from within the range of observed breast milk concentrations [18]. A recent trial in infants with CMPA fed an EHF supplemented with 2′-FL and LNnT suggested a protective effect against respiratory and ear infections [19]. A similar protective effect against lower respiratory tract infections and reduced antibiotic use had earlier been demonstrated in healthy infants fed a cow’s milk -based formula supplemented with the above two HMO [20].

Adequate growth and tolerability have previously been shown for an earlier version of the study formula without HMO [2,21]. The aim of the present study was to assess the growth, tolerability, and safety of the HMO-supplemented AAF in the target population of infants with moderate-to-severe CMPA. In addition, we aimed to explore the effects of the study formula on the developing gut microbiome.

## 2. Materials and Methods

The present study was conducted in six clinical sites in Australia between December 2018 and March 2021. Human ethics committee approval of the study protocol was obtained from the local institutional review boards at each of the participating clinical centers. Written informed consent was obtained from parents or legal guardians of the participating infants. The study was prospectively registered (ClinicalTrials.gov; NCT 03661736) and conducted in accordance with the principles and rules described in the Declaration of Helsinki and the international guideline on Good Clinical Practice (GCP).

### 2.1. Study Design

This single-arm, multicenter, interventional clinical trial was designed to assess the effects of a novel AAF supplemented with two HMO on growth, safety, and tolerability. Growth parameters were studied over a 4-month period (primary study endpoint). Infants were offered to remain on the study formula until 12 months of age (secondary study endpoint).

### 2.2. Inclusion and Exclusion Criteria

Term infants aged 1–8 months with physician-diagnosed moderate-to-severe CMPA were enrolled. All infants were non-breastfed at the time of enrollment. The diagnosis of moderate-to-severe CMPA required the fulfillment of at least one of the following criteria, in line with the clinical requirements for treatment with an AAF in the region: (1) severe IgE-mediated CMPA with previous anaphylaxis to cow’s milk protein (with a positive skin prick test ≥ 3 mm or cow’s milk-specific serum IgE ≥ 0.35 kUa/L); (2) IgE-mediated CMPA with a non-response to extensively hydrolyzed formula (EHF) due to immediate symptoms (urticaria, facial angioedema, vomiting, wheezing, or other respiratory distress); (3) moderate-to-severe gastrointestinal symptoms due to suspected non-IgE-mediated CMPA, not responding to a trial of EHF (persistent irritability/crying, persistent diarrhea with or without bright rectal bleeding, persistent constipation/fecal retention, persistent vomiting/regurgitation); (4) non-IgE-mediated multiple food protein intolerance of infancy (MFPI) with a non-response to a trial of EHF; (5) histologically proven eosinophilic esophagitis (EoE)–not previously treated with AAF or corticosteroids (presence of ≥15 eosinophils per microscopic high power field at 400 times magnification in upper and/or lower esophageal biopsies); or (6) infants previously or currently managed with an AAF or hydrolyzed rice-based formula (not supplemented with probiotics) for moderate-to-severe CMPA.

Infants were excluded from participating in the study if at least one of the following criteria was fulfilled: (1) known underlying medical condition that was likely to impair growth (e.g., unstable congenital heart disease, cystic fibrosis, metabolic disorder, chronic liver disease); (2) chronic malabsorption unrelated to CMPA; (3) other significant prenatal and/or serious postnatal disease; (4) infants receiving any breast milk at the time of enrollment; (5) treatment with systemic corticosteroids (oral or intravenous) for ≥72 h within 4 weeks prior to enrollment (topical corticosteroids allowed); (6) infants taking probiotic preparations, including probiotic-supplemented infant formulas, for ≥72 h within 4 weeks prior to enrollment; (7) parents or caregivers unable to give informed consent or deemed unable to comply with study procedures; or (8) Previous or current participation in another clinical trial.

### 2.3. Study Formula

The study formula (Alfamino, Nestlé Health Science, Switzerland) was an AAF for the management of infants with CMPA. The formula was supplemented with two HMO, 2′-FL and LNnT, at concentrations of 1.0 g/L and 0.5 g/L, respectively. The formula provided 66 kcal/100 mL of energy. The nitrogen source was based on free amino acids, equivalent to 2.66 g/100 kcal of protein (11.2% of total energy). The lipid blend (4.9 g/100 kcal; 45% of total energy) consisted of 24% of medium chain triglycerides (MCT), and the remainder included rapeseed oil, high oleic sunflower oil, re-esterified palm oil, docosahexaenoic acid (DHA), and arachidonic acid (ARA). In addition, the formula contained 11.3 g/100 kcal of carbohydrates from corn syrup (maltodextrin) and potato starch, but it was lactose-free. The study formula was nutritionally complete and suitable as a sole source of nutrition until 6 months of age. Parents or caregivers were asked to follow general feeding advice regarding appropriate feeding volumes per day, as printed on the formula label or as provided by their health care professional. A detailed summary of the nutrient composition of the study formula is provided in the online Supplementary Materials File S1.

### 2.4. Study Objectives and Endpoints

The primary objective of this clinical study was to assess the growth of infants with moderate-to-severe CMPA who were fed an AAF containing two HMO. The primary endpoint was weight gain from enrollment to 4 months of follow-up, compared with the WHO 2006 Child Growth Standard [22]. Secondary objectives included the assessment of linear growth (body length), head circumference (HC), and body mass index (BMI) from enrollment to the 4-month follow-up, as well as assessment of anthropometric parameters to 12 months of age (compared against the WHO 2006 Child Growth Standard). In addition, the study aimed to assess if the new infant formula was effective and safe in controlling the symptoms of infants with moderate-to-severe CMPA. Finally, changes in the composition of the gut microbiome and fecal short-chain fatty acids (SCFA) were characterized at several timepoints from enrollment to 12 months of age, with particular focus on the effects of the HMO-containing AAF on bifidobacteria and intestinal dysbiosis.

### 2.5. Monitoring of Symptom Resolution

Digestive tolerance and the alleviation of CMPA symptoms were evaluated by assessing infant behavior and symptoms (crying, fussing, spitting up, vomiting, feeding problems, skin symptoms, respiratory symptoms), as well as stool characteristics and frequency, at enrollment and on the 3 days preceding each study visit. Clinical details were recorded by parents in a diary. In addition, the medical investigator performed a physical examination and overall assessment at each visit based on parental report and diary information.

### 2.6. Schedule of Study Visits

Infants were fed the study formulas from enrollment (Visit 0; V0; ‘baseline’) to 4 months (V4) (principal study period). Demographic data, growth parameters, and other clinical information were documented at V0 and monitored monthly (V1, V2, V3, and V4). Families were offered to continue the study formula until 12 months of age, with a final follow-up visit at 12 months of age (V5).

### 2.7. Characterization of the Fecal Microbiome

Stool samples were collected at the baseline (V0) and follow-up visits V1, V4, and V5. Specimens were stored at −80 °C until processing. Microbial DNA was extracted from the frozen samples and sequenced, as previously described [23]. The Clinical Microbiomics in-house infant fecal microbiome gene catalog (containing 23,968,023 microbial genes) was used as the reference, and a set of 1306 corresponding metagenomic species (MGS) definitions applied for abundance profiling [24]. The taxonomical annotation was performed at the phylum, family, genus, species, and subspecies levels, based on the homology of the MGS catalog genes to the NCBI RefSeq genome database (published at https://www.ncbi.nlm.nih.gov/refseq; accessed on 27 January 2020). MGS composition, as well as alpha and beta diversity at baseline (V0) were compared to subsequent visits (V1, V4, and V5). Faith’s phylogenetic diversity (PD) index was used to assess alpha diversity [25]. The assessment of beta diversity was based on the weighted UniFrac distances for all visits (V0, V1, V4, and V5) [26].

As there was a considerable age range from 2 to 38 weeks at enrollment, the analysis was stratified by age group and visit. Differences in relative abundances of specific taxa between the study visits and three age windows (0–4 months, 4–6 months, and >6 months at enrollment) were evaluated at the phylum, family, genus, and species levels and compared by the Wilcoxon signed rank test, including false discovery rate correction (FDR; <0.1) for multiple comparisons. Eleven MGS annotated to the genus *Bifidobacterium* were included in the analysis: *B. breve:* MGS.hg0209, *B. longum* subsp. *infantis*: MGS.hg0464, *B. longum* subsp. *longum*: MGS.hg0021, *B. bifidum*: MGS.hg0100, *B. adolescentis*: MGS.hg0038, *B. dentium*: MGS.hg0537, *B. pseudocatenulatum*: MGS.hg0101, *B. catenulatum* subsp. *kashiwanohense*: MGS.hg0185, *B. angulatum*: MGS.hg0312, *B. animalis* subsp. *lactis*: MGS.hg0426, and *B. gallinarum*: MGS.ref1191. Of the above bifidobacteria, a set of six bifidobacteria (*B. bifidum*, *B. breve*, *B. longum* susp. *infantis*, *B. longum* subsp. *longum*, *B. pseudocatenulatum*, and *B. catenulatum* subsp. *kashiwahonense*) were grouped to assess changes in the relative abundances of HMO-utilizing bifidobacteria from the baseline to 12 months of age [10,27]. This included a set of four bifidobacteria (*B. breve*, *B. bifidum*, *B. longum* subsp. *infantis*, and *B. longum* subsp. *longum*) that were previously described as ‘infant-type’ by Laursen et al. [28]. The sum of the relative abundances of the HMO-utilizing set was compared between visits. This analysis was based on the hypothesis that the HMO-utilizing bifidobacteria were enriched at V1, V4, and V5 compared to V0. To test this hypothesis, we accepted statistical significance at *p* < 0.05 and did not apply the FDR correction for multiple comparisons.

Finally, a taxon set enrichment analysis (TSEA) was performed at the genus level to assess the changes of microbiota in relative abundance from the baseline (V0) to later visits (V1, V4, V5) [29]. The TSEA focused on the genus *Bifidobacterium* and several key genera, including *Bacteroides* spp., *Escherichia* spp., *Akkermansia* spp., as well as several butyrate- producing bacteria (e.g., *Faecalibacterium* spp., *Eubacterium* spp., *Roseburia* spp., and *Anaerostipes* spp.). A detailed description of the methods for the microbiome characterization is provided in the online Supplementary Materials File S2.

### 2.8. Measurement of Fecal Short Chain Fatty Acids

Fecal samples were stored at −80 °C until processed in the central reference laboratory. Concentrations of fecal acetate, propionate, and butyrate were measured by gas chromatography–mass spectrometry and reported in μmol per gram feces.

### 2.9. Adverse Event Reporting

Adverse events (AE) were reported by the investigators and coded for medical diagnosis, severity, and likely causality/relatedness to the study formula. AE were verified by an independent medical monitor.

### 2.10. Statistical Analysis

Demographics and anthropometric measurements were described by summary statistics (mean, standard deviation, median, interquartile range (IQR), percentage. Weight, length, and head circumference (HC) measurements were converted into weight-for-age (WAZ), length-for-age (LAZ), HC-for-age (HCAZ), and body mass index-for-age (BMIAZ) Z scores, according to the WHO child growth standards [22]. Changes in symptoms from V0 to subsequent visits were compared by χ^2^ analysis. Statistical significance was accepted for *p* < 0.05.

### 2.11. Determination of Sample Size

Given the descriptive nature of the study, the sample size was not determined by a formal power calculation. The enrollment of 62 infants was chosen as a meaningful sample size, with the aim of 50 infants completing the principal study period of 4 months (anticipated early withdrawal rate of 20%). The target number of enrolled subjects was later reduced to 30 completers due to difficulties in recruiting patients. Reasons included changes in the prescribing patterns for AAF in Australia, high rates of breast feeding, as well as a major negative impact of the COVID-19 pandemic on access to the six study sites during 2020. The enrollment of new subjects was halted in September 2020 after a prolonged period of non-recruitment.

## 3. Results

**Figure 1:**
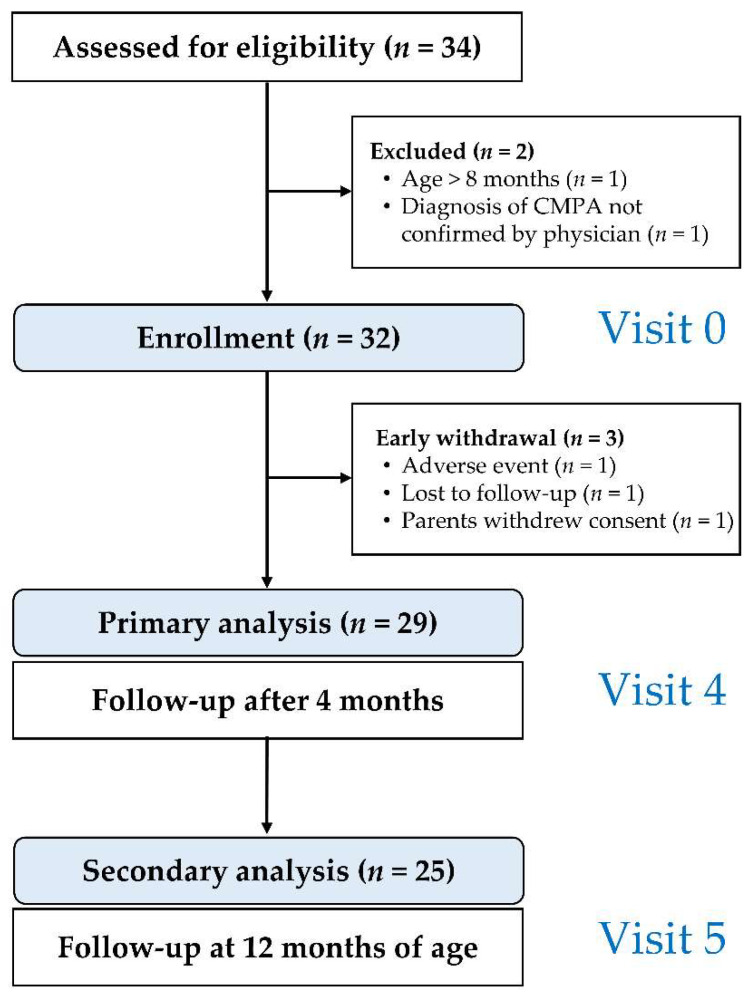
Study flow chart.

Out of the 34 infants screened, 32 were enrolled (mean age 18.6 weeks; range 4–37; 20 (62.5%) male). Twenty-nine infants completed the trial to the primary endpoint (V4). The study flow is summarized in Figure 1. Demographics and clinical details of infants at the time of enrollment are summarized in Table 1.

### 3.1. Clinical Presentation at Enrollment

All infants enrolled in the study had been diagnosed as suffering from CMPA by a pediatric allergist. The diagnosis was based on clinical symptoms alone in twenty-nine (90.6%) infants, and three (9.4%) infants also had a positive skin prick test to cow’s milk. None of the infants had undergone a formal open or double-blinded oral food challenge (OFC) at the time of enrollment. The clinical manifestations of CMPA are summarized in Table 2.

### 3.2. Previous Formula Use and Complementary Diet

Prior to commencing a hypoallergenic formula, infants had been trialed on several different types of infant formula by their parents: one formula in 12 (37.5%), two in 7 (21.9%), three in 10 (31.3%), and four different formulas in 3 (9.4%) infants. Twenty-two infants (68.8%) had been fed a cow’s milk-based formula, 27 (84.4%) a soy-based formula, 28 (87.5%) a partially hydrolyzed cow’s milk formula, and 23 (71.9%) a hydrolyzed rice-based formula (HRF). At the time of enrollment, 16 (50.0%) infants were being fed an EHF, 3 (9.4%) infants received an HRF, and 13 (40.6%) were being fed an AAF. Fifteen (46.9%) infants received a complementary weaning diet, which was commenced at a mean age of 4.43 ± 0.90 (SD) months.

### 3.3. Anthropometric Data

**Figure 2:**
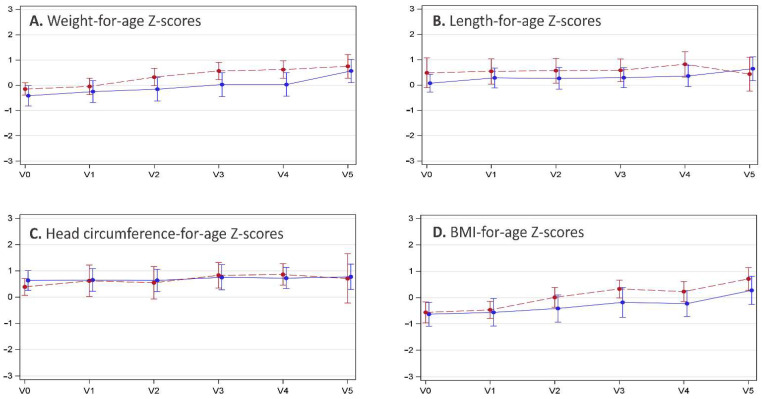
Anthropometric measurements are presented as mean Z-scores (WHO child growth standard) at enrollment (Visit 0, V0), the 1-month (V1), 2-month (V2), 3-month (V3), and 4-month follow-up visits, as well as the end-of-study visit at 12 months of age (V5). Error bars indicate the standard deviations. The blue dots/solid lines summarize the growth data for male infants, and red dots/broken lines summarize that for female infants. The four panels depict (**A**) weight-for-age, (**B**) length-for-age, (**C**) head circumference-for-age, and (**D**) body mass index (BMI)-for-age Z-scores.

Based on measurements from baseline to V4 (*n* = 28), the mean weight gain was 18.0 ± 6.13 g per day of formula intake (range 7.8–29.2 g/day). Z-scores for body weight from enrollment to V4 progressed close to the WHO child growth standard, with a minor upward trend towards the end of the first year of life (V5). The mean WAZ increased from −0.31 to +0.28 (delta WAZ +0.59), the LAZ from +0.23 to +0.55 (delta LAZ +0.32), and the HCAZ from +0.55 to +0.78 (delta HCAZ +0.23). The BMIAZ followed a similar pattern, with an increase from −0.61 to −0.04 (delta BMIAZ +0.57). The anthropometric measurements for male and female infants are summarized in Figure 2.

### 3.4. Formula Intake

The mean duration of formula administration for the principal study period from V0 to V4 was 122.2 ± 6.14 days, and 110.7 ± 47.01 days from V4 to the end of the study visit at V5. The mean formula intake from V0 to V1 was 822.2 ± 206.12 mL/day. The daily formula intake progressively decreased with each visit to 801.8 ± 161.48 mL/day at V2, 782.9 ± 159.70 mL/day at V3, 719.5 ± 200.48 mL/day at V4, and 558 ± 197.58 mL/day at V5 (12 months of age).

### 3.5. Resolution of CMPA Symptoms and Stool Characteristics

**Figure 3:**
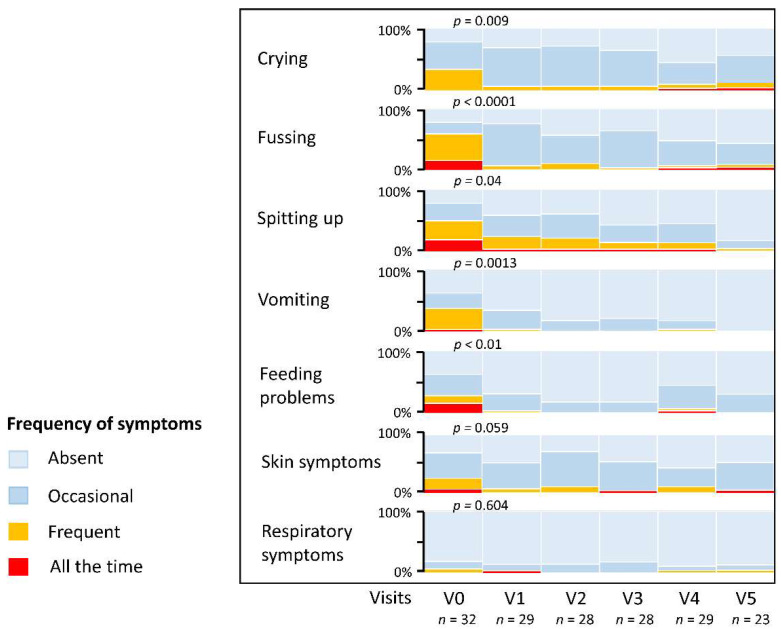
Prevalence of common symptoms from enrollment to 12 months of age. Symptoms were graded by the investigators as ‘absent’, ‘occasional’, ‘frequent’, or ‘all the time’. P-values are shown for the comparison of frequent and persistent symptoms at Visit 0 (V0) compared to Visit 1 (V1) after 1 month. V2, V3, and V4 were follow-up visits after 2, 3, and 4 months, respectively; the final study visit (V5) occurred at 12 months of age.

Symptoms improved significantly between enrollment (V0) and follow-up after one month’s treatment (V1). Comparing the proportion of infants with frequent or persistent (‘all the time’) symptoms, there was a 79.9% and 88.4% reduction in crying and fussing, respectively (χ^2^ = 6.745; *p* = 0.009). Similarly, the proportion of infants with frequent or persistent regurgitation (‘spitting up’) fell by 51.7% (χ^2^ = 4.274; *p* = 0.039), while vomiting was reduced by 90.8% (χ^2^ = 10.38; *p* = 0.0013). The prevalence of significant feeding difficulties at V1 was reduced by 87.7% (χ^2^ = 6.677; *p* = 0.010), and the prevalence of frequent or persistent skin problems fell from 25% to 6.9% (χ^2^ = 3.576; *p* = 0.059). Few infants had significant respiratory symptoms, with only two infants (6.3%) reporting frequent problems at enrollment. Persistent respiratory symptoms had resolved in one (3.4%) infant at V1 (χ^2^ = 0.269; *p* = 0.60). A summary of the prevalence of common symptoms is provided in Figure 3.

Regarding stool characteristics, there was a trend to more formed and less frequent stools with increasing age. At V1, the mean stool frequency was 1.5 ± 1.26 (range 0–6) bowel motions per day. At V4, the stool frequency was 1.6 ± 0.73 (range 0–4) and 1.4 ± 0.53 (range 0–2) at V5. Stool consistency was assessed by the Bristol stool scale (types 1–7) [30]. The median stool consistency decreased from Bristol type 6 stools (mushy consistency; interquartile range, IQR types 4–6) to type 5 stools (soft, semi-formed consistency; IQR 3.25–6) at V4, and type 4 stools (formed consistency; IQR types 3–5) at V5.

### 3.6. Safety

There were 232 adverse events (AE) in total, of which 192 (82.8%) were classified as mild, 37 (15.9%) as moderate, and 3 (1.3%) as severe. Six (18.8%) subjects experienced 8 serious adverse events (SAE), all of which were assessed as unrelated to the study formula. The majority of AE affected the gastrointestinal system (69 AE in 26 (81.3%) subjects). In addition, 84 adverse events due to infections were reported in 25 (78.1%) subjects. Fifteen (46.9%) subjects had 30 events due to skin disorders, mainly atopic dermatitis. Nine (28.1%) subjects experienced 12 events of respiratory disorders, and seven (21.9%) subjects had 13 allergic reactions to food (including one episode of anaphylaxis). Eight (25%) subjects reported 14 events due to general disorders or administration site conditions. The remaining 10 AE were coded as accidental injury (*n* = 2), accidental ingestion (*n* = 1), dacryostenosis (*n* = 1), poor feeding/poor weight gain (*n* = 2), perforated ear drum (*n* = 1), iron deficiency (*n* = 1), irritability (*n* = 1), and inadequate diet (*n* = 1).

Four AE in two (6.4%) subjects were deemed ‘related’ (*n* = 3) or ‘probably related’ (*n* = 1) and led to the discontinuation of the study formula in both cases. One of the subjects had presented with mild gastroesophageal reflux, and the other had developed loose stools, flatulence, and decreased feeding (all graded as mild).

### 3.7. Microbiological Analysis of Stool Samples

In total, 109 stool samples from 32 infants were available for DNA extraction and genomic sequencing from V0, V1, V4, and V5. All samples were of sufficient quality for genomic analysis. On average, 21.8 M read pairs per sample could be mapped to the reference gene catalog, representing on average 95.2% of the high-quality non-host reads (range 79.4–98.1%). Duplicate samples and samples of subjects only providing baseline samples were excluded, leaving 105 samples from 29 infants for the final analysis.

#### 3.7.1. Alpha and Beta Diversity

**Figure 4:**
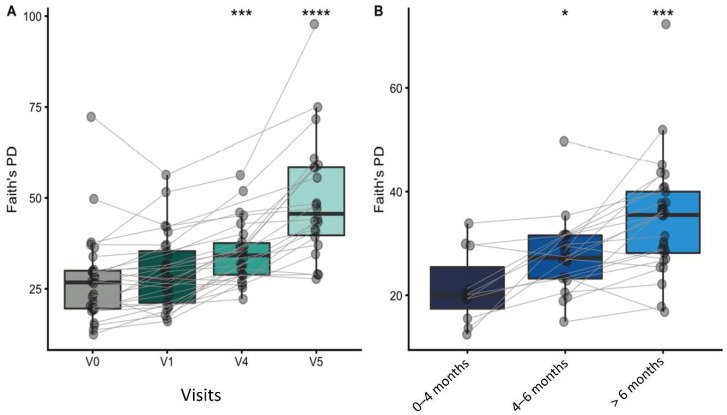
Box plots summarizing the changes in Faith’s phylogenetic diversity of gut microbiomes, grouped by study visit (**A**) and age window (**B**). Samples from the same infant are connected by lines. Paired group comparisons were made by Wilcoxon signed rank test between V0 and subsequent visits (**A**), as well as between infants enrolled at 0–4 months compared to older infants (**B**). Significant differences are indicated above the groups: * p < 0.05; *** *p* < 0.001; **** *p* < 0.0001.

Faith’s PD was calculated to assess the alpha diversity at each study visit. Overall, phylogenetic diversity increased with age. Faith’s PD increased significantly at V4 and V5, compared to the baseline (V0). Similarly, the PD increased significantly for each age group; Figure 4A,B.

**Figure 5:**
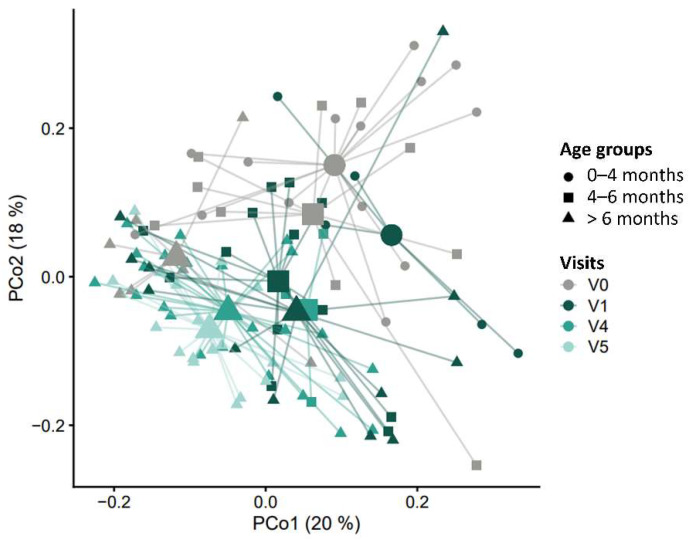
Beta diversity plot. The principal coordinates analysis (PCoA) shows weighted UniFrac distances among samples for study visits V0, V1, V4, and V5. The mean (centroid) of samples in each group is indicated with a larger shape. Each sample is connected to its centroid by a thin line. The *X*- and *Y*-axis labels indicate the microbial variance explained by the first two principal coordinates.

Changes in beta diversity, i.e., the overall microbiome community composition, were evaluated using weighted UniFrac distances, which reflect differences in relative abundance, as well as phylogenetic distances among MGS. There were highly significant group differences in beta diversity, both when comparing by study visits (R^2^ = 5.37%; *p* = 0.003) or age windows (R^2^ = 4.93%; *p* < 0.0001); Figure 5.

#### 3.7.2. Taxonomic Characterization and Temporal Development

**Figure 6:**
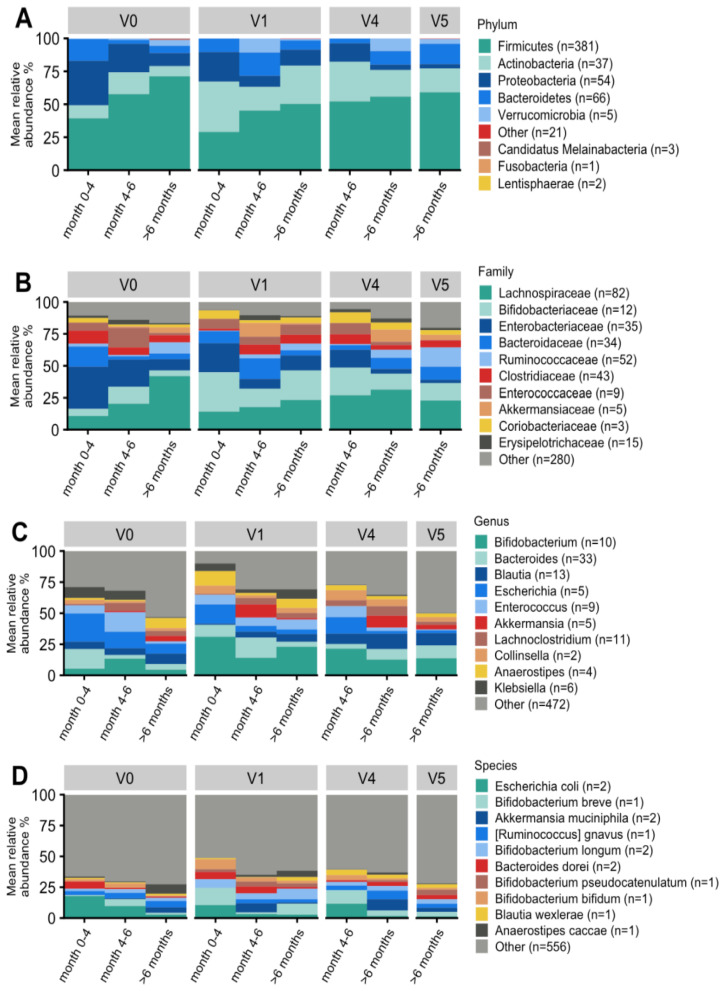
Taxonomic overview of the gut microbiome of infants with CMPA at the phylum (**A**), family (**B**), genus (**C**), and species (**D**) levels. Bar plots display the mean relative abundance within each visit and the age window of the top 10 taxa with the highest abundance. Gray (other) indicates the total relative abundance of MGS that could be classified but are not in the top 10 most abundant taxa. The number of MGS included in the aggregated taxon is shown in brackets.

The taxonomic analysis was performed using the metagenomic species (MGS) concept [24]. Figure 6 illustrates the taxonomic profiles aggregated at the phylum, family, genus, and species levels.

At the phylum level, there was a substantial enrichment in Actinobacteria (which includes bifidobacteria) from V0 to V1 (*p* = 0.01; FDR = 0.031), V4 (*p* = 0.001; FDR = 0.016), and V5 (*p* = 0.05; FDR = 0.071). Conversely, Proteobacteria were significantly reduced at V1 (*p* = 0.0006; FDR = 0.003), V4 (*p* < 0.00001; FDR < 0.00001), and V5 (*p* < 0.00001; FDR = 0.0004) (Figure 6A). Furthermore, at the family level, *Enterobacteriaceae* were highly abundant at V0 and significantly decreased at V1 (*p* = 0.0008; FDR = 0.022), V4 (*p* < 0.000001; FDR < 0.0001), and V5 (*p* < 0.0001; FDR < 0.0007) (Figure 6B).

At the genus level, the abundance of bifidobacteria increased with borderline significance from V0 to V1 (*p* = 0.016; FDR = 0.157) and V4 (*p* = 0.046; FDR = 0.137) (Figure 6C). The genus *Escherichia* spp. showed a highly significant reduction from V0 to V1 (*p* = 0.0002; FDR = 0.010), V4 (*p* < 0.00001; FDR < 0.00034), and V5 (*p* = 0.006; FDR = 0.030). The genus *Akkermansia* spp. Was enriched at V4 (*p* < 0.0031; FDR < 0.021) and borderline significant at V5 (*p* = 0.057; FDR = 0.121). The abundances of several genera were significantly increased at V5, compared to V0, including *Faecalibacterium* spp. (*p* = 0.0002; FDR = 0.0063), *Roseburia* spp. (*p* = 0.026; FDR = 0.068), *Bacteroides* spp. (*p* = 0.010; FDR = 0.034), and *Prevotella* spp. (*p* = 0.012; FDR = 0.036). Finally, the abundances of several genera were significantly decreased at V5 compared to V0, including *Rothia* spp. (*p* < 0.0001; FDR = 0.0002), *Klebsiella* spp. (*p* = 0.0009; FDR = 0.133), *Enterococcus* spp. (*p* = 0.0049; FDR = 0.025), *Streptococcus* spp. (*p* = 0.0071; FDR = 0.0303), and *Citrobacter* spp. (*p* = 0.009; FDR = 0.031).

Due to the relatively small sample size and significant age gradient at V0, there were few significant findings at the species level, and the comparisons of individual bifidobacterial species mostly did not reach statistical significance. *Escherichia coli* was significantly reduced at V1, V4, and V5 (*p* < 0.01; FDR < 0.05). In line with findings at the genus level, *Akkermansia muciniphila* was more abundant at V4 (*p* = 0.0046; FDR = 0.036) and borderline significant at V5 (*p* = 0.057; FDR = 0.130). Finally, butyrate-producing gut bacteria were significantly enriched, including *Faecalibacterium prausnitzii*, at V4 (*p* = 0.002; FDR = 0.025) and V5 (*p* = 0.0001; FDR = 0.0035), and *Anaerostipes caccae* (*p* = 0.001; FDR = 0.035) at V4 (Figure 6D).

#### 3.7.3. Effect on Bifidobacterial Composition and Abundances

Of the 11 selected MGS annotated to the genus *Bifidobacterium*, 10 were detected in at least one fecal sample. At V1, *B. breve*, *B. longum*, *B. pseudocatenulatum*, and *B. bifidum* were the most abundant bifidobacterial species (Figure 6D). When testing for differences in the relative abundance, *B. pseudocatenulatum*: MGS.hg0101 and *B. catenulatum* subsp. *kashiwanohense*: MGS.hg0185 were significantly increased at V1 (*p* = 0.047), V4 (*p* = 0.008) and V5 (*p* = 0.001), compared to V0. Furthermore, *the abundance of B. breve*: MGS.hg0209 increased from V0 to V1 with borderline statistical significance (*p* = 0.08).

**Figure 7:**
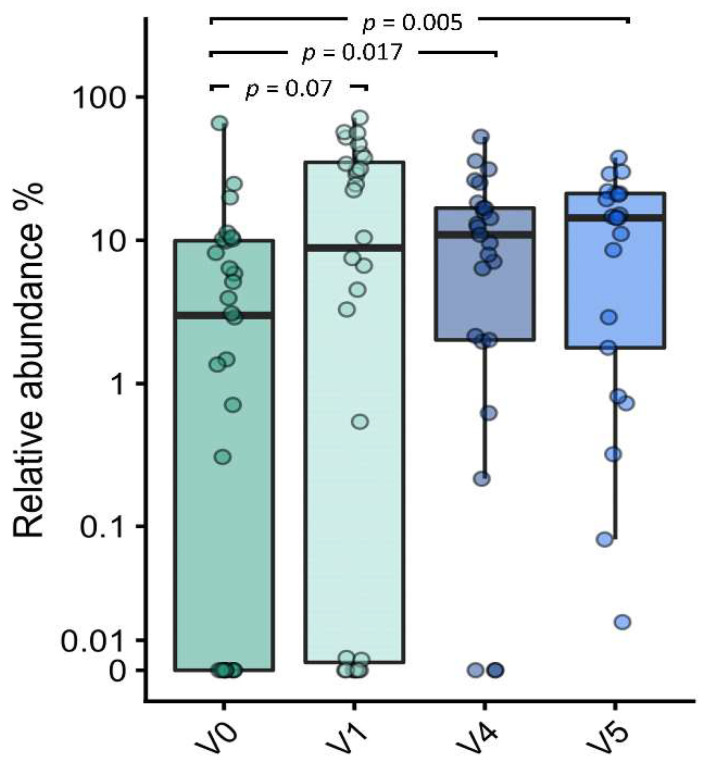
Boxplot of HMO-utilizing bifidobacteria at the baseline (V0) and follow-up visits at 1 month (V1), 4 months (V4), and 12 months of age (V5). Changes in relative abundance were assessed by the Wilcoxon signed rank test, comparing the baseline (V0) to subsequent visits.

A sub-analysis assessed the relative abundances of the HMO-utilizing *Bifidobacterium* set [10,27]. There was an increase of borderline significance in this set from V0 to V1 (*p* = 0.07), with a significant enrichment demonstrated for subsequent visits at V4 (*p* = 0.017) and V5 (*p* = 0.005) (Figure 7).

#### 3.7.4. Taxon Set Enrichment Analysis

The taxon set enrichment analysis (TSEA) was used to assess the enrichment or suppression of taxonomic groups based on changes in relative MGS abundances between visits. Table 3 shows the main significant findings of the TSEA at the genus level for V1, V4, and V5, compared to the baseline (V0).

**Figure 8:**
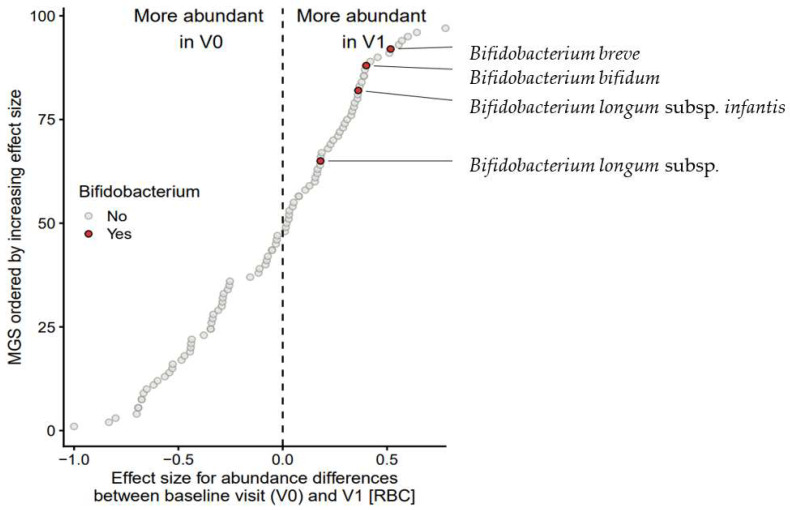
Taxon set enrichment analysis. Each circle represents the effect size (rank biserial correlation, RBC) of a single metagenomic species (MGS) when comparing the relative abundances between Visit 0 (V0) and Visit 1 (V1) in 28 infants with CMPA. The 97 included MGS (*Y*-axis) are sorted by their corresponding RBC-value (*X*-axis). Bifidobacterium (sub)species are highlighted in red.

At V1, four bifidobacterial species were significantly enriched (RBC = 0.704; *p* = 0.017; FDR = 0.083) (Table 3). These consisted of the infant-type bifidobacteria, *B. breve*, *B. bifidum*, *B. longum* subsp. *longum* and *B. longum* subsp. *infantis* (Figure 8). By contrast, the proteobacterium *Escherichia* spp. was significantly suppressed (RBC = –0.911; *p* = 0.007; FDR = 0.082). At V4, nine MGS from the genus *Lachnoclostridium* spp. were enriched (RBC = 0.707; *p* = 0.0005; FDR = 0.0006), while the Proteobacteria *Klebsiella* spp. (RBC = –0.671; *p* = 0.024; FDR = 0.065) and *Escherichia* spp. were suppressed (RBC = –0.994; *p* = 0.004; FDR = 0.020). At V5, six MGS of *Bacteroides* spp. were enriched (RBC = 0.911; *p* = 0.007; FDR = 0.082) at the expense of *Streptococcus* spp., *Escherichia* spp., and *Rothia* spp.; Table 3.

### 3.8. Fecal Short-Chain Fatty Acid Levels

**Figure 9:**
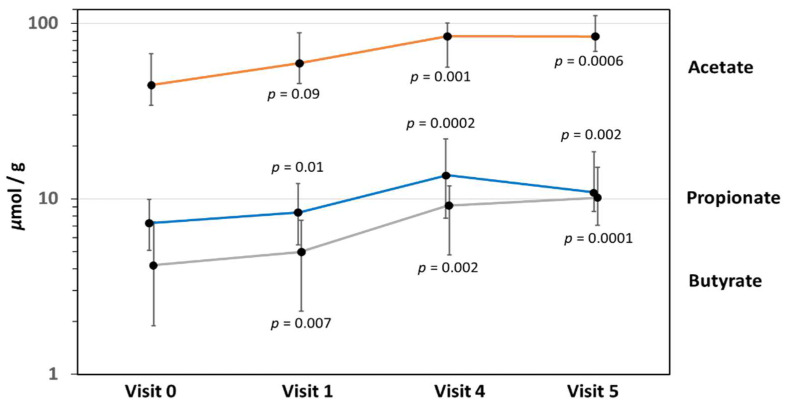
Mean fecal short-chain fatty acid concentrations for acetate, propionate, and butyrate (error bars indicate standard deviation). *p*-values are shown for the two-sample *t*-test comparing the baseline (V0) levels with those at the follow-up after 1 month (V1), 4 months (V4), and at 12 months of age (V5).

Fecal concentrations of the three SCFA, acetate, propionate, and butyrate, increased significantly from enrollment to 12 months of age (Figure 9). Relatively high levels of fecal acetate, a key metabolite of HMO-utilizing bifidobacteria, were found throughout the entire study to 12 months of age. Fecal butyrate levels increased progressively from V0 to V5, while propionate levels peaked at V4 and declined slightly towards V5.

## 4. Discussion

The present study assessed the growth, tolerability, and safety of a novel AAF supplemented with the HMO, 2′-FL and LNnT, in infants with moderate-to-severe CMPA. Over the principal 4-month intervention period, weight gain, linear growth, and head growth progressed along the WHO child growth standard, with a minor upward trend of about 0.5 standard deviations from the baseline. This pattern of accelerated growth is commonly seen in formula-fed infants, when compared to growth patterns of breastfed infants [31]. In addition, there may have been some catch-up growth in infants with pre-existing growth impairment due to CMPA. The mean weight gain in the present study was 18 g/day (range 7.8–29.2 g/day), which is lower than in an earlier growth study in healthy infants for the same AAF without HMO (27.42 ± 6.37 g/day) [2]. This difference is likely due to the higher age at enrollment of infants with CMPA (mean age 18 weeks in present study vs. 2 weeks in healthy infants), as well as possible growth retardation secondary to the effects of CMPA (e.g., malabsorption, regurgitation/vomiting, or poor feeding).

Infants enrolled into the present study had presented with a range of symptoms, mostly suggestive of non-IgE-mediated CMPA. Most participants had failed prior treatment with an EHF or hydrolyzed rice formula. Based on parent-report and physician assessment, symptoms improved significantly from enrollment to Visit 1, and further improvement in symptoms was observed at subsequent visits (Figure 3). Control of skin symptoms was generally excellent, with only one infant experiencing persistent skin problems to V5. Two infants discontinued the study formula due to mild adverse gastrointestinal symptoms, which were deemed related to the study formula.

As a secondary outcome, the study aimed to assess the changes in microbiome composition and fecal SCFA levels from enrollment to 12 months of age. The early development of the gut microbiome is a highly dynamic process that is influenced by birth method, diet, and environmental factors. However, by far the most important factor affecting the microbiome development in the first year of life is the type of feeding, i.e., breastfed infants have a significantly different microbiome and metabolome compared to formula-fed infants [32,33]. In the first months of life, HMO in breast milk provide the specific substrate for the establishment of a gut microbiome rich in infant-type bifidobacteria [7,34]. Not all bifidobacteria are able to assimilate HMO [10]. Several studies have suggested that infants acquire infant-type bifidobacteria from their mother via vertical transmission [35,36].

*Bifidobacterium* spp. generate a strict anaerobic milieu, and cessation of breastfeeding or of HMO supplementation leads to a sharp increase in Firmicutes and *Bacteroides* [32]. The colonization of the intestine with bifidobacteria provides significant benefits for early immune development [37]. Several HMO species have been shown to confer a protective effect against respiratory and gastrointestinal infections by interfering with glycan-binding and mucosal adhesion [20,38,39]. The beneficial effects of a bifidobacterium-rich microbiome appear to be related to several key metabolites, including SCFA [33,34]. A lack of bifidobacteria in early infancy and an overgrowth of Proteobacteria and other Gram-negative bacteria, also called ‘dysbiosis’, is thought to predispose to asthma and allergies in later childhood [40,41,42,43]. Supplementation with HMO may therefore potentially restore the abundance of bifidobacteria and counteract the effects of a dysbiotic microbiome [42]. This may be associated with health benefits, including a reduced risk for atopic manifestations.

In the present study, the gut microbiome of infants at the baseline had the hallmarks of dysbiosis, with a relative lack of bifidobacteria and an overgrowth of Proteobacteria and other gram-negative bacteria [43,44]. Following the change to the study formula, bifidobacteria increased significantly at V1. This increase was mainly seen for the HMO-utilizing bifidobacteria, suggesting a specific effect of 2′-FL and LNnT in the study formula [10,27]. The taxon set enrichment analysis confirmed the HMO-utilizing bifidobacteria, *B. breve*, *B. bifidum*, *B. longum* subsp. *infantis*, and *B. longum* subsp. *longum* were increased at V1. Although *B. pseudocatenolatum* and *B. catenulatum* subsp. *kashiwanohense* also were significantly enriched at V1, V4, and V5, the increase of these HMO-assimilating bifidobacteria was strongest at V5, suggesting a mixed HMO and diet effect [27].

Bifidobacteria produce acetate and other SCFA, which lower the colonic pH and create an acidic, protective milieu against enteropathogens [45]. In the present study, we found an overall increase in fecal SCFA from V0 to V5. High levels of fecal acetate throughout the study period point to the metabolic effects of bifidobacteria, in line with patterns seen in breastfed infants [33]. However, the absence of a control group did not allow to conclusively assess the relationship between fecal SCFA and HMO supplementation. The increase in SCFA may thus also have been affected by other dietary factors, particularly dietary fiber in the weaning diet [34]. Fecal SCFA have been shown to improve intestinal mucosal integrity, as well as colonic regulatory T cell homeostasis [46]. In the present study, only fecal SCFA levels were measured. We were therefore unable to directly correlate the fecal SCFA levels with clinical outcomes or perform a detailed assessment of the SCFA effect on immune function.

In recent years, the focus of the metabolic effects of bifidobacteria has moved beyond SCFA. HMO-degrading bifidobacteria express the enzyme aromatic lactate dehydrogenase (ALDH) and are able to produce aromatic lactic acids (indoleacetic acid, phenyllactic acid, and 4-hydroxyphenyllactic acid) from aromatic amino acids [28]. Aromatic lactic acids are thought to confer significant clinical benefits, including improved intestinal barrier function, protection from pathogenic infection, and effects on metabolic pathways [47,48,49]. Beneficial effects include the induction of immunoregulatory galectin-1 in T-helper 2 (Th2) and Th17 cells, which suggests a protective effect of HMO-utilizing bifidobacteria against allergic sensitization [11]. It therefore appears plausible that HMO supplementation and an increase in HMO-assimilating bifidobacteria would positively affect allergic outcomes.

Apart from the effect on bifidobacteria, the present study found other significant changes in bacterial species, which indicate a shift towards a healthy gut microbiome [50]. Overall, the abundance of Proteobacteria (including *Escherichia* spp., *Klebsiella* spp., and other potential pathogens) decreased from V0 to V1, suggesting a partial correction of the dysbiosis typically seen in infants with CMPA and other allergies [44]. The significant enhancement of *Bacteroides* from V0 to the end of the first year is typical of a healthy transition to an adult-type gut microbiome where *Bacteroides* play an important role in the colonic degradation of dietary fiber [9]. *Akkermansia muciniphila*, a functionally important Gram-negative bacterium related to mucus degradation was enriched at V4 and V5, compared to the baseline (Figure 6). A recent study has confirmed that *Akkermansia muciniphila* is able to utilize 2′-FL and other HMO [51], although this bacterium is more closely associated with complementary feeding [52]. Another important microbial species that was significantly enriched at V4 and V5 were butyrate-producing bacteria, including *Faecalibacterium prausnitzii* and *Anaerostipes caccae* [53,54]. Fecal numbers of *Faecalibacterium prausnitzii* are often undetectable before 6 months of age, but gradually increase until 2 years of age and peak in adolescence [55]. In infancy, acetate produced by bifidobacteria provides the substrate for the cross-feeding of butyrate producers [56,57]. In the present study, the emergence of butyrate producers was mirrored by the rise in fecal butyrate levels towards 12 months of age. Fecal butyrate is of great physiological importance for gut health and immune regulation and is reduced in infants with non-IgE-mediated CMPA [34,43,46].

The present study had several limitations. The diagnosis of CMPA was based on clinical symptoms and IgE-based testing, where available, but confirmatory diagnostic food challenges were not performed. The single-arm design of the study was another limitation, and the microbiome analysis was mainly descriptive, as a control group was not available for comparison. Therefore, the effects of HMO on the microbiome development could not be clearly differentiated against other influences, such as age, increasing dietary diversity, or fiber intake. In addition, the age range of 1–8 months at enrollment made it more difficult to differentiate HMO from age effect over the course of the intervention to 12 months of age. Finally, due to changes in prescription patterns for AAF in Australia and the COVID-19 epidemic with restricted access to hospital outpatient services, recruitment did not reach the target of 50 completers. The reduced sample size of 32 infants limited the statistical power for comparisons, which mainly affected the microbiome analysis. Despite these limitations, the study still provided useful clinical insights and supports the use of the study formula in infants with physician-diagnosed moderate-to-severe CMPA.

## 5. Conclusions

Infants with moderate-to-severe CMPA fed the study formula with two HMO achieved adequate growth, with some catch-up growth. The formula was tolerated well and had an excellent safety profile. The gut microbiome characterization demonstrated a significant early enrichment in HMO-utilizing, infant-type bifidobacteria, and later enrichment in *Bacteroides* and butyrate producing taxa in the second half of the first year. Conversely, there was a significant reduction in Proteobacteria, a marker phylum of gut dysbiosis. Microbiome changes were associated with a significant rise in fecal SCFA concentrations from enrollment to 12 months of age. These findings suggest that supplementation with 2′-FL and LNnT was associated with a significant enrichment in HMO-utilizing bifidobacteria and a partial correction the of the gut microbial dysbiosis in infants with CMPA. The clinical effects associated with the HMO-induced microbiome changes on immune maturation and tolerance development require further study.

## Figures and Tables

**Table 1:** Baseline characteristics of study participants who commenced treatment (*n* = 32).

Infant Characteristics	
Age, weeks ^1^	18.6 ± 8.02
Gestational age, weeks ^1^	38.6 ± 1.31
Male sex, *n* (%)	20 (62.5)
Birth by Cesarean section, *n* (%)	12 (37.5)
Race	
Caucasian/White, *n* (%)	29 (90.6)
Asian, *n* (%)	1 (3.1)
Australian Aboriginal or	1 (3.1)
Torres Strait Islander	
Unknown	1 (3.1)
Anthropometric measurements ^1^	
Weight, kg	6.5 ± 1.34
Length, cm	63.7 ± 4.70
Head circumference, cm	41.8 ± 2.42

^1^ Mean ± standard deviation.

**Table 2:** Clinical manifestations of CMPA at enrollment (*n* = 32).

Clinical Manifestations	*n* (%)
Urticaria	5 (15.6)
Angioedema	2 (6.3)
Anaphylaxis	0 (0)
Persistent crying/irritability	28 (87.5)
Frequent regurgitation/vomiting	20 (62.5)
Persistent diarrhea	19 (59.4)
Bright rectal bleeding	7 (21.9)
Constipation/fecal retention	3 (9.4)
Eczema/atopic dermatitis	17 (53.1)

**Table 3:** Summary of significant findings from the taxon set enrichment analysis (at genus level) performed on the associations of the relative abundances of metagenomic species (MGS) with visits.

Comparison	Genus	MGS ^1^ *n*	Enriched	RBC ^2^	*p* Value	FDR ^3^
V0 vs. V1	*Bifidobacterium* spp.	4	V1	0.704	0.017	0.083
	*Lachnoclostridium* spp.	9	V1	0.463	0.023	0.083
	*Escherichia* spp.	3	V0	−0.911	0.007	0.082
V0 vs. V4	*Lachnoclostridium* spp.	9	V4	0.706	0.0005	0.006
	*Klebsiella* spp.	4	V0	−0.671	0.024	0.065
	*Rothia* spp.	3	V0	−0.896	0.009	0.031
	*Escherichia* spp.	3	V0	−0.993	0.004	0.020
V0 vs. V5	*Bacteroides* spp.	6	V5	0.549	0.024	0.060
	*Streptococcus* spp.	3	V0	−0.565	0.020	0.060
	*Escherichia* spp.	3	V0	−0.809	0.017	0.060
	*Rothia* spp.	3	V0	−0.975	0.004	0.041

^1^ MGS—metagenomic species; ^2^ RBC—rank biserial correlation; ^3^ FDR—false discovery rate <0.1 (adjusted for multiple comparisons).

## Data Availability

Data are available on request from the Chief Science and Medical Officer, Nestlé Health Science, 1800 Vevey, Switzerland.

## Supplementary Materials

The following supporting information can be downloaded at: https://www.mdpi.com/article/10.3390/nu14112297/s1, File S1: Nutrient composition of the study formula; File S2: Detailed Methods for Gut Microbiome Characterization.

## References

[1] Meyer R., Groetch M., Venter C. (2018). When Should Infants with Cow’s Milk Protein Allergy Use an Amino Acid Formula? A Practical Guide. J. Allergy Clin. Immunol. Pract..

[2] Corkins M., Czerkies L.A., Storm H.M., Sun S., Saavedra J.M. (2016). Assessment of Growth of Infants Fed an Amino Acid-Based Formula. Clin. Med. Insights Pediatr..

[3] Muraro A., Werfel T., Hoffmann-Sommergruber K., Roberts G., Beyer K., Bindslev-Jensen C., Cardona V., Dubois A., duToit G., Eigenmann P. (2014). EAACI food allergy and anaphylaxis guidelines: Diagnosis and management of food allergy. Allergy.

[4] Fiocchi A., Brozek J., Schünemann H., Bahna S.L., von Berg A., Beyer K., Bozzola M., Bradsher J., Compalati E., Ebisawa M. (2010). World Allergy Organization (WAO) Diagnosis and Rationale for Action against Cow’s Milk Allergy (DRACMA) Guidelines. World Allergy Organ. J..

[5] Kemp A.S., Hill D.J., Allen K.J., Anderson K., Davidson G.P., Day A.S., Heine R.G., Peake J.E., Prescott S.L., Shugg A.W. (2008). Guidelines for the use of infant formulas to treat cows milk protein allergy: An Australian consensus panel opinion. Med. J. Aust..

[6] Rachid R., Chatila T.A. (2016). The role of the gut microbiota in food allergy. Curr. Opin. Pediatr..

[7] Borewicz K., Gu F., Saccenti E., Hechler C., Beijers R., de Weerth C., van Leeuwen S.S., Schols H.A., Smidt H. (2020). The association between breastmilk oligosaccharides and faecal microbiota in healthy breastfed infants at two, six, and twelve weeks of age. Sci. Rep..

[8] Asakuma S., Hatakeyama E., Urashima T., Yoshida E., Katayama T., Yamamoto K., Kumagai H., Ashida H., Hirose J., Kitaoka M. (2011). Physiology of consumption of human milk oligosaccharides by infant gut-associated bifidobacteria. J. Biol. Chem..

[9] Marcobal A., Sonnenburg J.L. (2012). Human milk oligosaccharide consumption by intestinal microbiota. Clin. Microbiol. Infect..

[10] Sakanaka M., Gotoh A., Yoshida K., Odamaki T., Koguchi H., Xiao J.Z., Kitaoka M., Katayama T. (2019). Varied Pathways of Infant Gut-Associated Bifidobacterium to Assimilate Human Milk Oligosaccharides: Prevalence of the Gene Set and Its Correlation with Bifidobacteria-Rich Microbiota Formation. Nutrients.

[11] Henrick B.M., Rodriguez L., Lakshmikanth T., Pou C., Henckel E., Arzoomand A., Olin A., Wang J., Mikes J., Tan Z. (2021). Bifidobacteria-mediated immune system imprinting early in life. Cell.

[12] Zuurveld M., van Witzenburg N.P., Garssen J., Folkerts G., Stahl B., Van’t Land B., Willemsen L.E.M. (2020). Immunomodulation by Human Milk Oligosaccharides: The Potential Role in Prevention of Allergic Diseases. Front. Immunol..

[13] Dogra S.K., Martin F.P., Donnicola D., Julita M., Berger B., Sprenger N. (2021). Human Milk Oligosaccharide-Stimulated Bifidobacterium Species Contribute to Prevent Later Respiratory Tract Infections. Microorganisms.

[14] Vandenplas Y., Berger B., Carnielli V.P., Ksiazyk J., Lagstrom H., Sanchez Luna M., Migacheva N., Mosselmans J.M., Picaud J.C., Possner M. (2018). Human Milk Oligosaccharides: 2’-Fucosyllactose (2’-FL) and Lacto-N-Neotetraose (LNnT) in Infant Formula. Nutrients.

[15] Wicinski M., Sawicka E., Gebalski J., Kubiak K., Malinowski B. (2020). Human Milk Oligosaccharides: Health Benefits, Potential Applications in Infant Formulas, and Pharmacology. Nutrients.

[16] Faijes M., Castejon-Vilatersana M., Val-Cid C., Planas A. (2019). Enzymatic and cell factory approaches to the production of human milk oligosaccharides. Biotechnol. Adv..

[17] Kunz C., Rudloff S. (2017). Compositional Analysis and Metabolism of Human Milk Oligosaccharides in Infants. Nestle Nutr. Inst. Workshop Ser..

[18] Liu S., Cai X., Wang J., Mao Y., Zou Y., Tian F., Peng B., Hu J., Zhao Y., Wang S. (2021). Six Oligosaccharides’ Variation in Breast Milk: A Study in South China from 0 to 400 Days Postpartum. Nutrients.

[19] Vandenplas Y., Żołnowska M., Berni Canani R., Ludman S., Tengelyi Z., Moreno-Álvarez A., Goh A.E.N., Gosoniu M.L., Kirwan B.A., Tadi M. (2022). Effects of an Extensively Hydrolyzed Formula Supplemented with Two Human Milk Oligosaccharides on Growth, Tolerability, Safety and Infection Risk in Infants with Cow’s Milk Protein Allergy: A Randomized, Multi-Center Trial. Nutrients.

[20] Puccio G., Alliet P., Cajozzo C., Janssens E., Corsello G., Sprenger N., Wernimont S., Egli D., Gosoniu L., Steenhout P. (2017). Effects of Infant Formula With Human Milk Oligosaccharides on Growth and Morbidity: A Randomized Multicenter Trial. J. Pediatr. Gastroenterol. Nutr..

[21] Vandenplas Y., Dupont C., Eigenmann P., Heine R.G., Høst A., Järvi A., Kuitunen M., Mukherjee R., Ribes-Koninckx C., Szajewska H. (2021). Growth in infants with cow’s milk protein allergy fed an amino acid-based formula. Pediatr. Gastroenterol. Hepatol. Nutr..

[22] World Health Organization (2006). WHO Child Growth Standards: Length/Height-for-Age, Weight-for-Age, Weight-for-Length, Weight-for-Height and Body Mass Index-for-Age: Methods and Development.

[23] Hauser J., Pisa E., Arias Vasquez A., Tomasi F., Traversa A., Chiodi V., Martin F.P., Sprenger N., Lukjancenko O., Zollinger A. (2021). Sialylated human milk oligosaccharides program cognitive development through a non-genomic transmission mode. Mol. Psychiatry.

[24] Nielsen H.B., Almeida M., Juncker A.S., Rasmussen S., Li J., Sunagawa S., Plichta D.R., Gautier L., Pedersen A.G., Le Chatelier E. (2014). Identification and assembly of genomes and genetic elements in complex metagenomic samples without using reference genomes. Nat. Biotechnol..

[25] Faith D.P., Baker A.M. (2007). Phylogenetic diversity (PD) and biodiversity conservation: Some bioinformatics challenges. Evol. Bioinform. Online.

[26] Hamady M., Lozupone C., Knight R. (2010). Fast UniFrac: Facilitating high-throughput phylogenetic analyses of microbial communities including analysis of pyrosequencing and PhyloChip data. ISME J..

[27] Ojima M.N., Asao Y., Nakajima A., Katoh T., Kitaoka M., Gotoh A., Hirose J., Urashima T., Fukiya S., Yokota A. (2022). Diversification of a Fucosyllactose Transporter within the Genus Bifidobacterium. Appl. Environ. Microbiol..

[28] Laursen M.F., Sakanaka M., von Burg N., Morbe U., Andersen D., Moll J.M., Pekmez C.T., Rivollier A., Michaelsen K.F., Molgaard C. (2021). Bifidobacterium species associated with breastfeeding produce aromatic lactic acids in the infant gut. Nat. Microbiol..

[29] Pedersen H.K., Forslund S.K., Gudmundsdottir V., Petersen A.O., Hildebrand F., Hyotylainen T., Nielsen T., Hansen T., Bork P., Ehrlich S.D. (2018). A computational framework to integrate high-throughput ‘-omics’ datasets for the identification of potential mechanistic links. Nat. Protoc..

[30] Lewis S.J., Heaton K.W. (1997). Stool form scale as a useful guide to intestinal transit time. Scand. J. Gastroenterol..

[31] Agostoni C., Grandi F., Gianni M.L., Silano M., Torcoletti M., Giovannini M., Riva E. (1999). Growth patterns of breast fed and formula fed infants in the first 12 months of life: An Italian study. Arch. Dis. Child..

[32] Stewart C.J., Ajami N.J., O’Brien J.L., Hutchinson D.S., Smith D.P., Wong M.C., Ross M.C., Lloyd R.E., Doddapaneni H., Metcalf G.A. (2018). Temporal development of the gut microbiome in early childhood from the TEDDY study. Nature.

[33] Bridgman S.L., Azad M.B., Field C.J., Haqq A.M., Becker A.B., Mandhane P.J., Subbarao P., Turvey S.E., Sears M.R., Scott J.A. (2017). Fecal Short-Chain Fatty Acid Variations by Breastfeeding Status in Infants at 4 Months: Differences in Relative versus Absolute Concentrations. Front. Nutr..

[34] Riviere A., Selak M., Lantin D., Leroy F., De Vuyst L. (2016). Bifidobacteria and Butyrate-Producing Colon Bacteria: Importance and Strategies for Their Stimulation in the Human Gut. Front. Microbiol..

[35] Milani C., Mancabelli L., Lugli G.A., Duranti S., Turroni F., Ferrario C., Mangifesta M., Viappiani A., Ferretti P., Gorfer V. (2015). Exploring Vertical Transmission of Bifidobacteria from Mother to Child. Appl. Environ. Microbiol..

[36] Makino H., Kushiro A., Ishikawa E., Muylaert D., Kubota H., Sakai T., Oishi K., Martin R., Ben Amor K., Oozeer R. (2011). Transmission of intestinal *Bifidobacterium longum* subsp. *longum* strains from mother to infant, determined by multilocus sequencing typing and amplified fragment length polymorphism. Appl. Environ. Microbiol..

[37] Gensollen T., Iyer S.S., Kasper D.L., Blumberg R.S. (2016). How colonization by microbiota in early life shapes the immune system. Science.

[38] Etzold S., Bode L. (2014). Glycan-dependent viral infection in infants and the role of human milk oligosaccharides. Curr. Opin. Virol..

[39] Andersson B., Porras O., Hanson L.A., Lagergard T., Svanborg-Eden C. (1986). Inhibition of attachment of *Streptococcus pneumoniae* and *Haemophilus influenzae* by human milk and receptor oligosaccharides. J. Infect. Dis..

[40] Stokholm J., Blaser M.J., Thorsen J., Rasmussen M.A., Waage J., Vinding R.K., Schoos A.M., Kunoe A., Fink N.R., Chawes B.L. (2018). Maturation of the gut microbiome and risk of asthma in childhood. Nat. Commun..

[41] Joseph C.L., Sitarik A.R., Kim H., Huffnagle G., Fujimura K., Yong G.J.M., Levin A.M., Zoratti E., Lynch S., Ownby D.R. (2022). Infant gut bacterial community composition and food-related manifestation of atopy in early childhood. Pediatr. Allergy Immunol..

[42] Kumar H., Collado M.C., Wopereis H., Salminen S., Knol J., Roeselers G. (2020). The Bifidogenic Effect Revisited-Ecology and Health Perspectives of Bifidobacterial Colonization in Early Life. Microorganisms.

[43] Berni Canani R., De Filippis F., Nocerino R., Paparo L., Di Scala C., Cosenza L., Della Gatta G., Calignano A., De Caro C., Laiola M. (2018). Gut microbiota composition and butyrate production in children affected by non-IgE-mediated cow’s milk allergy. Sci. Rep..

[44] Rachid R., Stephen-Victor E., Chatila T.A. (2021). The microbial origins of food allergy. J. Allergy Clin. Immunol..

[45] Walsh C., Lane J.A., van Sinderen D., Hickey R.M. (2020). Human milk oligosaccharides: Shaping the infant gut microbiota and supporting health. J. Funct. Foods.

[46] Smith P.M., Howitt M.R., Panikov N., Michaud M., Gallini C.A., Bohlooly Y.M., Glickman J.N., Garrett W.S. (2013). The microbial metabolites, short-chain fatty acids, regulate colonic Treg cell homeostasis. Science.

[47] Zelante T., Iannitti R.G., Cunha C., De Luca A., Giovannini G., Pieraccini G., Zecchi R., D’Angelo C., Massi-Benedetti C., Fallarino F. (2013). Tryptophan catabolites from microbiota engage aryl hydrocarbon receptor and balance mucosal reactivity via interleukin-22. Immunity.

[48] Dodd D., Spitzer M.H., Van Treuren W., Merrill B.D., Hryckowian A.J., Higginbottom S.K., Le A., Cowan T.M., Nolan G.P., Fischbach M.A. (2017). A gut bacterial pathway metabolizes aromatic amino acids into nine circulating metabolites. Nature.

[49] Krishnan S., Ding Y., Saedi N., Choi M., Sridharan G.V., Sherr D.H., Yarmush M.L., Alaniz R.C., Jayaraman A., Lee K. (2018). Gut Microbiota-Derived Tryptophan Metabolites Modulate Inflammatory Response in Hepatocytes and Macrophages. Cell Rep..

[50] Wilmanski T., Rappaport N., Diener C., Gibbons S.M., Price N.D. (2021). From taxonomy to metabolic output: What factors define gut microbiome health?. Gut Microbes.

[51] Kostopoulos I., Elzinga J., Ottman N., Klievink J.T., Blijenberg B., Aalvink S., Boeren S., Mank M., Knol J., de Vos W.M. (2020). *Akkermansia muciniphila* uses human milk oligosaccharides to thrive in the early life conditions in vitro. Sci. Rep..

[52] Li N., Liang S., Chen Q., Zhao L., Li B., Huo G. (2021). Distinct gut microbiota and metabolite profiles induced by delivery mode in healthy Chinese infants. J. Proteom..

[53] Nilsen M., Saunders C.M., Angell I.L., Arntzen M.O., Carlsen K.C.L., Carlsen K.H., Haugen G., Hagen L.H., Carlsen M.H., Hedlin G. (2020). Butyrate Levels in the Transition from an Infant- to an Adult-Like Gut Microbiota Correlate with Bacterial Networks Associated with *Eubacterium rectale* and *Ruminococcus gnavus*. Genes.

[54] Pryde S.E., Duncan S.H., Hold G.L., Stewart C.S., Flint H.J. (2002). The microbiology of butyrate formation in the human colon. FEMS Microbiol. Lett..

[55] Miquel S., Martin R., Bridonneau C., Robert V., Sokol H., Bermudez-Humaran L.G., Thomas M., Langella P. (2014). Ecology and metabolism of the beneficial intestinal commensal bacterium *Faecalibacterium prausnitzii*. Gut Microbes.

[56] Duncan S.H., Barcenilla A., Stewart C.S., Pryde S.E., Flint H.J. (2002). Acetate utilization and butyryl coenzyme A (CoA):Acetate-CoA transferase in butyrate-producing bacteria from the human large intestine. Appl. Environ. Microbiol..

[57] Chia L.W., Mank M., Blijenberg B., Bongers R.S., van Limpt K., Wopereis H., Tims S., Stahl B., Belzer C., Knol J. (2021). Cross-feeding between *Bifidobacterium infantis* and *Anaerostipes caccae* on lactose and human milk oligosaccharides. Benef. Microbes.

