# Supplementary material for: Effects of an Amino Acid-Based Formula Supplemented with Two Human Milk Oligosaccharides on Growth, Tolerability, Safety, and Gut Microbiome in Infants with Cow’s Milk Protein Allergy
Source: Nutrients. 2022 May 30;14(11):2297. doi: 10.3390/nu14112297 (PMC9182596; doi:10.3390/nu14112297)
Supplement: Supplementary file 1 [file nutrients-14-02297-s001.zip › supplementary/Supplementary materials S1.pdf]

Nutrient Product Declaration

ALFAMINO®, Nestlé Health Science, Switzerland

Composition of study formula for Platypus Trial (Australia, 2017)

Nutrients	Unit	per 100g	per 100kcal	per 1000 mL serving
Energy	kcal	**499.80**	**100.00**	**664.28**
Energy	kJ	**2091.16**	**418.40**	**2779.34**
Water	g	**2.80**	**0.56**	**902.10**
Protein	g	**13.30**	**2.66**	**17.68**
Fat	g	**24.60**	**4.94**	**32.69**
Available Carbohydrates	g	**56.30**	**11.27**	**74.83**
Sum of Fibers	g	**1.00**	**0.20**	**1.33**
Lacto-N-neotetraose	g	**0.38**	**0.08**	**0.50**
2'-O-Fucosyllactose	g	**0.76**	**0.15**	**1.00**
C22:6 n-3 Docohexaenoic Acid (DHA)	mg	**175.00**	**35.01**	**232.59**
C18:3 n-3 Alpha-Linolenic Acid	g	**0.49**	**0.90**	**0.60**
C20:4 n-6 Arachidonic Acid	mg	**175.00**	**35.01**	**232.59**
C18:2 n-6 Linoleic Acid	g	**4.00**	**0.80**	**5.32**
Medium Chain Triglycerides	g	**6.00**	**1.20**	**7.98**
Sum Monounsaturated Fatty Acids	g	**9.50**	**1.90**	**12.63**
Sum Polyunsaturated Fatty Acids	g	**4.50**	**0.90**	**5.98**
Sum Saturated Fatty Acids	g	**9.00**	**1.80**	**11.96**
Lactose	g	**<0.05**	**<0.01**	**<0.066**
Sugars	g	**4.00**	**0.80**	**5.32**
Ca (Calcium)	mg	**530.00**	**106.52**	**704.37**
Mg (Magnesium)	mg	**45.00**	**9.04**	**59.81**
P (Phosphorus)	mg	**350.00**	**70.35**	**465.15**
Na (Sodium)	mg	**180.00**	**36.01**	**239.24**
K (Potassium)	mg	**570.00**	**114.05**	**757.58**
Cl (Chloride)	mg	**420.00**	**84.03**	**558.22**

Nutrients	Unit	per 100g	per 100kcal	per 1000 mL serving
Fe (Iron)	mg	**5.00**	**1.00**	**6.65**
Cu (Copper)	mg	**0.40**	**0.08**	**0.53**
Zn (Zinc)	mg	**5.00**	**1.00**	**6.65**
Se (Selenium)	µg	**27.00**	**5.40**	**35.89**
Mn (Manganese)	µg	**90.00**	**18.01**	**119.62**
I (Iodine)	µg	**120.00**	**24.01**	**159.49**
Vitamin A	µgRE	**560.00**	**110.04**	**731.00**
Total Vitamin D [D2+D3]	µgD	**12.00**	**2.40**	**15.95**
Vitamin E	mgTE	**9.20**	**1.84**	**12.23**
Vitamin K1 (Phytomenadione)	µg	**45.00**	**9.00**	**59.81**
Vitamin C	mg	**80.00**	**16.01**	**106.33**
Vitamin B1	mg	**0.50**	**0.10**	**0.67**
Vitamin B2	mg	**1.00**	**0.20**	**1.33**
Niacin	mg	**6.40**	**1.28**	**8.51**
Niacin Equivalent	mg	**11.80**	**2.37**	**15.68**
Vitamin B6	mg	**0.40**	**0.08**	**0.53**
Total Folic Acid	µg	**70.00**	**14.01**	**93.04**
Total Folate Equivalent (DFE)	µg DFE	**116.67**	**23.34**	**155.06**
Pantothenic Acid	mg	**3.20**	**0.64**	**4.25**
Vitamin B12	µg	**1.55**	**0.31**	**2.06**
Biotin	µg	**11.60**	**2.32**	**15.42**
Choline	mg	**145.00**	**29.01**	**192.71**
Inositol	mg	**35.00**	**7.00**	**46.52**
Taurine	mg	**40.00**	**8.00**	**53.16**
L-Carnitine	mg	**8.50**	**1.70**	**11.30**
