# Supplementary material for: Effects of an Amino Acid-Based Formula Supplemented with Two Human Milk Oligosaccharides on Growth, Tolerability, Safety, and Gut Microbiome in Infants with Cow’s Milk Protein Allergy
Source: Nutrients. 2022 May 30;14(11):2297. doi: 10.3390/nu14112297 (PMC9182596; doi:10.3390/nu14112297)
Supplement: Supplementary file 1 [file nutrients-14-02297-s001.zip › supplementary/Supplementary materials S2.pdf]

SUPPLEMENTARY MATERIALS (S2)

Detailed Methods for Gut Microbiome Characterization

1. DNA Extraction and Sequencing

DNA extraction and sequencing was performed as described previously [1]. In short, microbial DNA was extracted and purified from frozen feces using the NucleoSpin Soil kit (Macherey-Nagel, Dueren, Germany). Lysis was performed by bead beating horizontally on a Vortex-Genie 2 at 2700 rpm for 5 minutes. Library preparation and next-generation sequencing were performed at Novogene (Cambridge, UK). The purified genomic DNA was randomly sheared into fragments of ~350 base pairs (bp) and used for library construction using the NEBNext Ultra Library Prep Kit for Illumina (New England Biolabs, Ipswich, USA). Libraries were evaluated using Qubit 2.0 fluorometer (Thermo Fisher Scientific, Waltham, USA) quantitation and Agilent 2100 Bioanalyzer (Agilent, Santa Clara, USA) for the fragment size distribution. Quantitative real-time PCR was used to determine the concentration of the final library prior to sequencing. Paired-end 2×150 bp sequencing was performed on an Illumina NovaSeq 6000 (Illumina, San Diego, USA).

2. Data Pre-processing

Raw FASTQ files were filtered to remove human genomic contamination by discarding read pairs mapped to the human reference genome GRCh38 with Bowtie2 (v. 0.2.3.4.1) [2]. Reads were then trimmed to remove adapters and bases with a Phred score below 20 using AdapterRemoval (v. 2.2.4) [3].

3. Gene Catalog and Metagenomic Species Definitions

The Clinical Microbiomics in-house infant fecal microbiome gene catalog (containing 23,968,023 microbial genes) was used as reference gene catalog, and the corresponding set of 1,306 metagenomics species (MGS) definitions for abundance profiling. The MGS were built based on >5000 deep-sequenced human adult and infant gut samples using an approach based on the metagenomic species concept [4] and have highly coherent abundance and base composition in a set of 1,776 independent reference human gut samples.

4. Mapping Reads to Gene Catalog

Trimmed, human-filtered reads were mapped to the gene catalog using BWA mem (v. 0.7.16a) [5]. An individual read was considered mapped to a gene if the mapping quality (MAPQ) was ≥20 and the read aligned with ≥95% identity over ≥100 bp. However, if >10 bases of the read did not align to the gene or extend beyond the gene, the read was considered unmapped. Reads meeting these criteria except for the MAPQ threshold were considered multi-mapped. Each read pair was counted as either 1) mapped to a specific gene, if one or both individual reads mapped to a gene, or 2) multi-mapped, if neither read was mapped, and at least one was multi-mapped, or 3) unmapped, if neither individual read mapped. If the two reads each mapped to a different gene, the gene mapped by read 1 was counted but not the gene mapped by read 2. The resulting gene count table, of number of mapped read pairs for each gene, was used to calculate the relative abundance of each MGS.

5. Taxonomical Annotation of MGS

MGSs were annotated by blasting catalog genes to the NCBI RefSeq genome database (2020-01-27) using a minimum of 80% sequence coverage with varying levels of similarity: 95, 95, 85, 75, 65, 55, 50, and 45% for gene taxonomy annotation at

subspecies, species, genus, family, order, class, phylum, and superkingdom level, respectively. To assign species, genus, family, order, class, and phylum or superkingdom level taxonomy to an MGS, we required 75, 60, 50, 40, 30, and 25%, respectively, of its genes to be consistently annotated to the same taxa at the given level. Furthermore, for species and at genus level annotation, we required that less than 10% of the remaining MGS genes to be annotated to any alternative taxon. Finally, we applied CheckM to each MGS [6], and updated our annotations with CheckM annotation for 118 MGSs for which CheckM provided annotation at higher resolution (lower taxonomic rank).

6. Relative Abundance Calculation of MGS

For each MGS, a signature gene set had been previously defined as the 100 genes optimized for accurate abundance profiling of the MGS. An MGS count table was created by counting the number of reads mapped to the MGS signature genes per sample. An MGS was considered detected if reads from a sample mapped to at least three of its signature genes; measurements that did not satisfy this criterion were set to zero. Based on internal benchmarks, this threshold results in 99.6% specificity. The MGS count table was normalized according to effective gene length and then normalized sample-wise to sum to 100%, resulting in relative abundance estimates for each MGS.

7. Ecological Measures

All alpha and beta diversity calculations were based on down-sampled (rarefied) MGS abundance profiles to control for uneven sampling. These were calculated by random sampling, without replacement, of a fixed number of signature gene counts per sample, and then following the procedure described above.

A phylogenetic tree connecting the MGS was generated using previously identified conserved genes. The species tree for the MGS was created based on single-copy bacterial and archaeal marker genes from the Genome Taxonomy Database (GTDB) consisting of 120 bacterial and 122 archaeal marker-genes belonging to either TIGRFAM or PFAM protein families [7, 8]. First, INTERPROSCAN [9] was used to identify marker genes within each MGS. Multi-copy marker genes and marker genes that were showed in < 10 MGS were excluded, resulting in a total of 111 bacterial and 26 archaeal marker genes with sufficient coverage. Seven of the 130 marker genes were shared between bacteria and archaea. MGS with fewer than 10 marker genes identified by this method and MGS that were annotated as eukaryotes were excluded. Protein sequences from these 130 marker genes were aligned using HMMalign (v.3.2.1), and non-aligned residues were trimmed from the multiple sequence alignment. The species tree was next inferred using the concatenation-based species tree approach in IQ-tree [10] with 1000 ultrafast bootstraps202F [11] and an edge-linked partition model [12]. The species tree covered a total of 1,255 MGS and was rooted with archaea as an outgroup. Faith's phylogenetic diversity (PD) [13] and weighted UniFrac distances [14] were calculated using this tree with the *PhyloMeasures* and *phyloseq* R packages, respectively.

8. Taxon Set Enrichment Analysis

Taxon set enrichments was performed at the genus level, as follows: Only MGS detected with abundance >0.01% in at least 10% of the samples in either of the visits compared were considered [15]. For these MGS, a Mann Whitney-U test of relative abundances was performed, and MGS were sorted on the rank-biserial correlation (RBC) of the test such that genera that are more abundant in one visit were at the top

of the list, and vice versa. Adequate statistical power was assured by filtering out genera with too few MGS to provide a False Discovery Rate (FDR) correction. For each remaining genus, performed a Mann-Whitney U test, comparing the ranks of MGS belonging to the genus to the ranks of MGS not belonging to the genus.

9. Statistics

For the weighted UniFrac analysis, permutational multivariate analysis of variance (PERMANOVA) tests assessing marginal effects of the terms were performed using the adonis2 function from the *vegan* R package with 1000 permutations [16]. Alpha diversity indexes and taxonomical abundances were compared among the groups using Wilcoxon signed rank test. All tests were performed as paired comparisons. All statistical tests were run using R software (v. 4.0.3). The relative abundances were compared between visits at the phylum, family genus, species level. For the first four levels the MGS-level abundances were aggregated (summed). Abundances were compared pairwise by Wilcoxon signed rank test between baseline (Visit 0; V0) and follow-up visits after 1 month (V1), after 4 months (V4), as well as at 12 months of age (V5). The analysis was corrected for multiple comparisons by applying an FDR<0.1.

For the *Bifidobacterium* analysis, 6 of the selected 11 MSG were grouped the as HMO-utilizing bifidobacteria: *B. bifidum, B. breve, B. longum* subsp. *infantis*, and *B. longum* subsp. *longum, B. pseudocatenulatum* and *B. catenulatum* subsp. *kashiwanohense* [17, 18]. This set included four 'infant-type' bifidobacteria (*B. longum* subsp. *infantis, B. longum* subsp. *longum, B. bifidum*, and *B. breve),* as described by Laursen *et al.* [19]. The sum of the relative abundances of the HMO-utilizing set was compared between visits. This analysis was based on the hypothesis that HMO-utilizing bifidobacteria were enriched at V1, V4 and V5, compared to V0. To test this hypothesis, we accepted statistical significance at p<0.05 and did not apply the FDR correction for multiple comparisons.

References

1. Hauser J, Pisa E, Arias Vasquez A, Tomasi F, Traversa A, Chiodi V, Martin FP, Sprenger N, Lukjancenko O, Zollinger A, et al. Sialylated human milk oligosaccharides program cognitive development through a non-genomic transmission mode. *Mol Psychiatry* **2021**, *26*, 2854-2871.
2. Langmead B, Salzberg SL. Fast gapped-read alignment with Bowtie 2. *Nat Methods* **2012**, *9*, 357-359.
3. Schubert M, Lindgreen S, Orlando L. AdapterRemoval v2: rapid adapter trimming, identification, and read merging. *BMC Res Notes* **2016**, *9*, 88.
4. Nielsen HB, Almeida M, Juncker AS, Rasmussen S, Li J, Sunagawa S, Plichta DR, Gautier L, Pedersen AG, Le Chatelier E, et al. Identification and assembly of genomes and genetic elements in complex metagenomic samples without using reference genomes. *Nat Biotechnol* **2014**, *32*, 822-828.
5. Li H, Durbin R. Fast and accurate short read alignment with Burrows-Wheeler transform. *Bioinformatics* **2009**, *25*, 1754-1760.
6. Parks DH, Imelfort M, Skennerton CT, Hugenholtz P, Tyson GW. CheckM: assessing the quality of microbial genomes recovered from isolates, single cells, and metagenomes. *Genome Res* **2015**, *25*, 1043-1055.
7. Parks DH, Chuvochina M, Chaumeil PA, Rinke C, Mussig AJ, Hugenholtz P. A complete domain-to-species taxonomy for Bacteria and Archaea. *Nat Biotechnol* **2020**, *38*, 1079-1086.
8. Parks DH, Chuvochina M, Waite DW, Rinke C, Skarshewski A, Chaumeil PA, Hugenholtz P. A standardized bacterial taxonomy based on genome phylogeny substantially revises the tree of life. *Nat Biotechnol* **2018**, *36*, 996-1004.
9. Jones P, Binns D, Chang HY, Fraser M, Li W, McAnulla C, McWilliam H, Maslen J, Mitchell A, Nuka G, et al. InterProScan 5: genome-scale protein function classification. *Bioinformatics* **2014**, *30*, 1236-1240.
10. Nguyen LT, Schmidt HA, von Haeseler A, Minh BQ. IQ-TREE: a fast and effective stochastic algorithm for estimating maximum-likelihood phylogenies. *Mol Biol Evol* **2015**, *32*, 268-274.
11. Hoang DT, Chernomor O, von Haeseler A, Minh BQ, Vinh LS. UFBoot2: Improving the Ultrafast Bootstrap Approximation. *Mol Biol Evol* **2018**, *35*, 518-522.
12. Chernomor O, von Haeseler A, Minh BQ. Terrace Aware Data Structure for Phylogenomic Inference from Supermatrices. *Syst Biol* **2016**, *65*, 997-1008.
13. Faith DP, Baker AM. Phylogenetic diversity (PD) and biodiversity conservation: some bioinformatics challenges. *Evol Bioinform Online* **2007**, *2*, 121-128.
14. Hamady M, Lozupone C, Knight R. Fast UniFrac: facilitating high-throughput phylogenetic analyses of microbial communities including analysis of pyrosequencing and PhyloChip data. *ISME J* **2010**, *4*, 17-27.
15. Pedersen HK, Forslund SK, Gudmundsdottir V, Petersen AO, Hildebrand F, Hyotylainen T, Nielsen T, Hansen T, Bork P, Ehrlich SD, et al. A computational framework to integrate high-throughput '-omics' datasets for the identification of potential mechanistic links. *Nat Protoc* **2018**, *13*, 2781-2800.
16. Anderson MJ, Ellingsen KE, McArdle BH. Multivariate dispersion as a measure of beta diversity. *Ecol Lett* **2006**, *9*, 683-693.
17. Sakanaka M, Gotoh A, Yoshida K, Odamaki T, Koguchi H, Xiao JZ, Kitaoka M, Katayama T. Varied Pathways of Infant Gut-Associated Bifidobacterium to Assimilate Human Milk Oligosaccharides: Prevalence of the Gene Set and Its Correlation with Bifidobacteria-Rich Microbiota Formation. *Nutrients* **2019**, *12*.
18. Ojima MN, Asao Y, Nakajima A, Katoh T, Kitaoka M, Gotoh A, Hirose J, Urashima T, Fukiya S, Yokota A, et al. Diversification of a Fucosyllactose Transporter within the Genus Bifidobacterium. *Appl Environ Microbiol* **2022**, *88*, e0143721.
19. Laursen MF, Sakanaka M, von Burg N, Morbe U, Andersen D, Moll JM, Pekmez CT, Rivollier A, Michaelsen KF, Molgaard C, et al. Bifidobacterium species associated with breastfeeding produce aromatic lactic acids in the infant gut. *Nat Microbiol* **2021**, *6*, 1367-1382.
